# Effect of Wheatgrass Juice on Nutritional Quality of Apple, Carrot, Beet, Orange and Lemon Juice

**DOI:** 10.3390/foods11030445

**Published:** 2022-02-02

**Authors:** Sanja Grubišić, Marija Kristić, Miroslav Lisjak, Katarina Mišković Špoljarić, Sonja Petrović, Sonja Vila, Andrijana Rebekić

**Affiliations:** 1 Faculty of Agrobiotechnical Sciences Osijek, Josip Juraj Strossmayer University of Osijek, Vladimira Preloga 1, 31000 Osijek, Croatia; sgrubisic@fazos.hr (S.G.); mkristic@fazos.hr (M.K.); mlisjak@fazos.hr (M.L.); spetrovic@fazos.hr (S.P.); sonja.vila@fazos.hr (S.V.); 2 Faculty of Medicine, Josip Juraj Strossmayer University of Osijek, Josipa Huttlera 4, 31000 Osijek, Croatia; kmiskovic@mefos.hr

**Keywords:** wheatgrass, in vitro bioaccessibility, juice, macro-elements, microelements, antioxidative potential

## Abstract

Fresh fruit and vegetable juices are commonly consumed as a valuable source of nutrients, while wheatgrass juice is, due to its nutritional value, used as a natural dietary supplement. The main aim of this research was to evaluate the effect of wheatgrass juice addition to apple, beet, carrot, orange, and lemon juice on total and in vitro bioaccessible concentrations of K, Ca, Mg, Mn, Fe, and Zn, vitamin C concentration, total phenolic and flavonoid content, and antioxidant activity. In comparison to other juices, wheatgrass juice had the highest total and in vitro bioaccessible concentrations of Ca, Mg, Mn, Fe, and Zn, while beet juice had the highest K concentration. Lemon and orange juices had the highest vitamin C concentration, while the highest total phenolic and flavonoid content were found in wheatgrass juice. After the addition of wheatgrass juice, Ca, Mg, Mn, and Zn concentration increased in all examined juices, vitamin C concentration increased in apple, beet, and carrot juice, total phenolic content increased in carrot juice, while total flavonoid content increased in apple, carrot, and orange juice. In comparison to the examined juices, wheatgrass juice has better nutritional value, and it could be used in a mixture with other juices to improve their nutritional value.

## 1. Introduction

A sedentary lifestyle, an accelerated pace of life, a lack of a balanced diet and physical activity, and access to fast foods rich in fats and sugars have contributed to the growing incidence of obesity in humans. Globally, obesity is a major public health problem as it leads to the development of further health problems, such as type 2 diabetes, cardiovascular disease, breast and colon cancer. In children and adolescents, overweight and obesity can also cause serious illness, which can later affect the quality of life and life expectancy. In 2016, more than 1.9 billion adults over the age of 18 were overweight, while 650 million were obese, and over 340 million children and adolescents between the ages of 5 and 19 were overweight or obese [1]. Since being overweight and obese can be prevented, education about a healthy and balanced diet is extremely important. Due to the high content of minerals, vitamins, and fiber, fresh fruits and vegetables play a basic role in a balanced diet. In addition, they are characterized by low calories and fat. Numerous studies have shown that diets rich in fruits and vegetables are associated with a lower risk of cardiovascular disease, stroke, and cancer and increased longevity. Due to its practicality, it is becoming popular to consume daily amounts of fruits and vegetables in the form of fresh juice. Furthermore, consuming fresh juice makes it easier to absorb nutrients than consuming solid foods, and it has been found that consuming them stimulates the activity of the digestive system.

Wheatgrass is a young shoot of wheat (*Tritium aestivum* L.), which is used as a natural dietary supplement due to its high nutritional value. Wheatgrass can be consumed in three different forms, such as fresh juice, powder, or tablets. It has been determined that wheatgrass has a rich mineral (Ca, P, Mg, K, Fe, Zn, B, Mo) and vitamin (A, C, E, and B complex) composition. It is also characterized by a high content of bioflavonoids, a wide number of enzymes, and all nine essential amino acids [2]. Due to the high content of chlorophyll, wheatgrass can be used for detoxifying the body and lowering blood pressure. Wheatgrass juice has been shown to significantly affect the treatment of patients with anemia [3] and thalassemia [4]. The reason for this is that chlorophyll and hemoglobin have an identical molecular structure, with the difference in the central atom. In chlorophyll, the central atom is magnesium, while in hemoglobin, it is iron [5]. In addition, research has shown that wheatgrass helps in the treatment of various health problems, such as periodontitis [6], ulcerative colitis [7], and it also helps in treating oncology patients [8,9,10].

When it comes to the nutrient composition of foods, especially in fruits and vegetables, the amount of the total mineral content is undoubtedly important information. In addition to the total content, it is important to know how much of the total mineral content is available for absorption into the organism or to know the bioaccessibility of nutrients [11]. Bioaccessibility can be determined by in vivo and in vitro models. Due to the extreme complexity of the digestive system, in vitro models may not be as accurate as in vivo, but they are faster and cheaper and can provide important information for further research of bioaccessibility using in vivo models [12,13].

Many studies have been carried out on fruit and vegetable juices as well wheatgrass juice nutritional value [14,15], but what is known little about is how wheatgrass juice affects the nutritional value of different fruit and vegetable juices. According to our knowledge, this is the first research that assesses the effect of WGJ addition on the nutritional value of other fruit and vegetable juices.

Accordingly, the purpose of this study is to examine the vitamin C, total phenolic and flavonoid content, antioxidant activity, and the total and in vitro bioaccessible concentrations of macro- and microelements in fresh fruit and vegetable juices that are commonly used in households and in fresh wheatgrass juice. In addition, since many find the taste and smell of wheatgrass juice not appealing for consumption, the study also examined the effect of the addition of wheatgrass and lemon juice to other juice. The main idea of this research is that the addition of wheatgrass juice to other juices will increase their nutritional value, while on the other hand, the unappealing smell and taste of fresh wheatgrass juice will be neutralized at the same time. Furthermore, the addition of lemon juice to other fruit and vegetable juices was tested with an aim to evaluate how it affects the bioaccessibility of microelements.

## 2. Materials and Methods

### 2.1. Plant Material

In this study, three types of fruits juices (apple, orange, and lemon), two types of vegetable juices (carrots, beets), and wheatgrass juice (*Triticum aestivum* L.) were examined. To examine the effect of wheatgrass juice (WGJ) and lemon juice on the nutritional value of other juices, each juice was mixed with wheatgrass juice and lemon juice, as is shown in Table 1.

Apples, oranges, lemons, carrots, and beets were bought in a local supermarket, while wheatgrass was cultivated in controlled conditions in the Laboratory of Plant Genetics and Biotechnology at the Faculty of Agrobiotechnical Sciences in Osijek. Winter wheat genotype “Ilirija” was used for the production of wheatgrass. Preparation of seeds and sowing was completed as is described in Grubisic et al. (2019) [5]. Wheatgrass was grown in a plant growth chamber for twelve days. A growing condition was set as follows: day/night cycle 14/10 and temperature was 22 °C during the day and 20 °C during the night. On the twelfth day after sowing, wheatgrass leaves were cut with ordinary alcohol sterilized scissors. Wheatgrass juice was made using a BL—30 hand juicer (Be Lih Do Enterprise Co., Ltd., Taoyuen, Taiwan). Apple, carrot, and beetroot juice were made using a home electric juicer, while orange and lemon juices were made using an ordinary citrus strainer. All juices were made fresh on the day of the simulation of in vitro digestion, while the 1 mL of juice for determination of total mineral concentrations was stored in clean plastic Falkon tubes that were frozen in an ultra-cold freezer at −80 °C until the day of the analysis.

### 2.2. Simulation of In Vitro Digestion

Simulation of in vitro digestion followed the method according to Minekus et al. (2014) [16], with certain modifications. This method proposed was a standardized method in which it was possible to simulate three phases of the digestive system (oral phase, gastric phase, and intestinal phase). Since liquid samples (juices) were used, the oral phase was omitted in this study, and the protocol was followed by the digestion gastric and intestinal phase. In the fresh juice samples of Simulated Gastric Fluid (SGF), the enzymes pepsin (Merck KGaA, Darmstadt, Germany) and CaCl_2_ were added. In the gastric phase, the pH of the sample should be 2.5, so the pH was reduced with 1 M HCl. The samples were placed in an aqueous bath with a shaker (GFL 1092, Burgwedel, Germany) for incubation at 200 rpm at a temperature of 37 °C for two hours, during which the pH was checked after one hour. At the end of the gastric phase, samples were taken from the water bath and prepared for the intestinal phase. In the samples, simulated intestinal fluids (SIF), enzymes, pancreatin (Merck KGaA, Darmstadt, Germany), and bile salts (Merck KGaA, Darmstadt, Germany), CaCl_2_, were added, and the pH was raised to 6.5 with 1 M NaOH. The samples were returned to a water bath with a shaker for incubation at 200 rpm at a temperature of 37 °C for two hours. After one hour, the pH of the samples was checked. After completion of the intestinal phase, the samples were placed on ice for 5 min and centrifuged at 4000 rpm at 4 °C for 15 min (Centrifuge Eppendorf 5810, Hamburg, Germany). After centrifugation, the supernatant was transferred into plastic Falcon 15 mL tubes and stored in an ultra-cold freezer at −80 °C until the measurement of mineral concentrations.

### 2.3. Determination of Total Concentrations of Macro and Microelements

Total and bioaccessible concentrations of macro- (K, Ca, and Mg) and microelements (Fe, Zn and, Mn) were measured on ICP–OES device (Perkin Elmer–Optima 2100 DV, Überlingen, Germany). Prior to measurement, fresh juice samples were wet digested with 9 mL 65% (*v*/*v*) HNO3 and 2 mL 30% (*v*/*v*) H2O2 in microwave vessels CEM Mars 6 (Matthews, NC, USA) according to [17]. In vitro, bioaccessible concentrations were measured directly in samples that were processed through the simulation of in vitro digestion.

The percentage of bioaccessibility (% B) of the tested elements was calculated as the ratio of the concentration in the samples after the in vitro digestion (B) and the total concentration (U) as follows: % B = B/U × 100.

### 2.4. Total Phenols and Flavonoids Content

To detect the total content of phenols and flavonoids, 0.1 mL of juice was extracted with 1 mL of 70% ethanol for 48 h at −20 °C. Flavonoids were detected according to [18] after the specific reaction of 200 µL of plant extract with AlCl_3_. Absorbance measured at 415 nm was compared with the absorbance of a range of standard quercetin (QC) solutions containing 0–20 µg QC mL^−1^. Results are expressed as µg QC mL^−1^ juice. Phenols were detected according to [19]. The concentration of blue complex with Folin–Ciocalteu reagent was measured spectrophotometrically (Varian Cary 50 UV-VIS, Agilent Technologies, Inc., Santa Clara, CA, USA) at 765 nm and compared with the absorbance of standard gallic acid (GA) solutions containing 0.0–0.0003 µg GA mL^−1^. Results are expressed as µg GA mL^−1^ juice.

### 2.5. Free Radical Scavenging by DPPH

The free radical-scavenging activity of juices was measured using the method described by [20], with some modifications. The juice was extracted with 70% ethanol. Sample stock solutions (0.5 g mL^−1^) were diluted to final concentrations of 40, 60, 80, 100 mg mL^−1^ in ethanol. The decrease in absorbance at 517 nm was measured spectrophotometrically (Varian Cary 50 UV-VIS, Agilent Technologies, Inc., Santa Clara, CA, USA) after 30 min incubation in the dark in the supernatants, to which was added 0.04% DPPH/ethanol solution. The generated curve was used to calculate 50% inhibition of the DPPH reagent decomposition reaction (IC50).

### 2.6. Ascorbic Acid Content

Ascorbic acid content was determined spectrophotometrically (Varian Cary 50 UV-VIS, Agilent Technologies, Inc., Santa Clara, CA, USA) at 520 nm according to [21], with modification. Juice (0.3 mL) was extracted with distilled water. By adding 13.3% trichloroacetic acid 2% dinitrophenylhydrazine-thiourea-copper sulfate reagent on sample extracts, ascorbic acid was transferred to a red bis-hydrazone during incubation time 3 h on 37 °C. After incubation, 65% sulfuric acid was added. The calibration curve was delineated using the ascorbic acid solution as a standard. The concentration of vitamin C is expressed in mg 100 mL^−1^ juice.

### 2.7. Statistical Analysis

The experiment was carried out as a completely randomized design with three replicates. Statistical analyses were performed using the Enterprise Guide software, version 8.3 (SAS Institute Inc., Cary, NC, USA). The Kolmogorov–Smirnov normality test (*p* < 0.05) was used to test if the variable follows a normal distribution. A difference between the means of total and bioaccessible macro- and microelements concentrations was tested using analysis of variance (ANOVA) at the significance level of *p* < 0.01, followed by Tukey’s Honestly Significant Difference (HSD) test. A difference between the means of the chlorophyll, vitamin C, total phenols and flavonoids, and antioxidant activity were tested by Kruskal-Wallis test (*p* < 0.05), adjusted by Bonferroni correction for multiple tests.

## 3. Results and Discussion

### 3.1. Concentrations of Macro and Microelements in Fresh Fruit, Vegetable, and Wheatgrass Juice

#### 3.1.1. Differences in Total and In Vitro Bioaccessible Concentrations of Macro- and Microelements in Different Fruit and Vegetable Juices

Three fruit (apple, orange, and lemon), two vegetable (beet and carrot) juices, and WGJ were examined with an aim to compare macro- (K, Ca, and Mg) and microelement (Fe, Mn, Zn) concentrations in fresh juices. In general, all juices had the highest total and in vitro bio-accessible concentrations of K, followed by Mg and Ca (Table 2). Significant differences between juices were found in K, Mg, and Ca totals and in vitro bioaccessible concentrations (Table 2).

The highest K total and in vitro bioaccessible concentrations were found in beet juice, followed by WGJ, which had a 20% lower total and 28% lower in vitro bioaccessible concentration in comparison to beet juice. In other fruit and vegetable juices, K concentration was lower than in WGJ. Furthermore, WGJ had the highest total and in vitro bioaccessible Ca and Mg concentrations (Table 2) in comparison to other juices. For example, WGJ had a 4.78-fold higher total Ca concentration in comparison to orange juice, which had the second-highest Ca concentration. Among fruit and vegetable juices, orange and lemon had the highest Ca, while beet and orange had the highest Mg concentration. The lowest concentrations of K, Ca, and Mg was found in fresh apple juice (Table 2). Based on that, WGJ can be considered a potent source of K, Ca, and Mg. Similar mineral concentrations and relations between minerals in wheatgrass are found in [22]. On the other hand, low K, Ca, and Mg concentrations in other juices could be due to different growing conditions, post-harvest, and storage treatment [23]. Yet, since most people buy fruits and vegetables and cannot influence the method of cultivation and storage conditions, it is assumed that the purchased fruits and vegetables will have similar mineral concentrations and generally lower element concentrations than fresh wheatgrass.

As for microelements, WGJ had the highest Mn, Fe, and Zn concentration compared to other juices (Table 3) and similar to concentrations obtained in [22]. The largest differences between WGJ and other juices were found for Mn concentration, where juices had between 9-fold (beet and carrot) to 64-fold (lemon juice) less Mn than WGJ. In beet juice, Fe concentration was 34% lower, while Zn concentration was only 2% lower than in WGJ. Carrot, orange, and lemon juices had much lower Fe concentrations than WGJ (65%, 45%, and 92%, respectively). Similar results were found for Zn concentration, where carrot (71%), orange (88%), and lemon (80%) juices had significantly lower Zn concentrations than WGJ (Table 3).

In vitro bioaccessible concentration represents the amount of the element that is potentially available for absorption in the organism [24]. When it is put in relation to a total concentration of the same element, we obtain a percentage of bioaccessibility. Based on that percentage, the bioaccessibility of different elements can be compared. In this research, K had the highest percentage of bioaccessibility in comparison to Ca and Mg, ranging from 73% in apple and orange to 96% in beet juice. Such a high percentage of bioaccessibility indicates that beet juice is a good source of K since 96% of the total K, which is found in the juice, could be absorbed in organisms. On the other hand, beet juice had the lowest Ca percentage of bioaccessibility (12%), while the highest percentage of bioaccessibility for Ca was found in WGJ (71%). A percentage of Mg bioaccessibility was in a range between 52% (apple juice) and 76% (beet and WGJ).

In general, the percentage of bioaccessibility of microelements was lower than in macro-elements. In WGJ, the highest percentage of bioaccessibility was obtained for Mn (77%), followed by Fe (57%) and Zn (38%). However, in other juices, the percentage of bioaccessibility did not follow this ordering. For example, orange juice had the highest Zn (94%) percentage of bioaccessibility and the lowest Fe (17%) bioaccessibility, while in beet juice percentage of bioaccessibility decreased in the following order Zn (52%), Mn (42%), Fe (18%). In carrot juice, total concentrations were low, but in vitro bioaccessible concentrations were high compared to other juices, resulting in a high percentage of bioaccessibility (Mn 50%, Fe 73%, and Zn 73%). Many factors affect the bioavailability and absorption of minerals in the human organism [25], but the most important among them are chemical form, concentration, interactions between nutrients and antinutrients. Antinutrients are bioactive compounds that interfere absorption of nutrients. Some well-known antinutrients are phytic acid, oxalates, lectins, and trypsin inhibitors [26]. Zn, Fe, Mg, and Ca bioavailability can be decreased by the presence of phytic acid [27].

#### 3.1.2. The Effect of WGJ Addition on Total and In Vitro Bioaccessible Concentrations of Macro- and Microelements in Different Fruit and Vegetable Juices

Consumption of food supplements is on a constant rise. Among supplements that are most often used are multivitamins, minerals (Ca, Mg, Fe, Zn), proteins, fish oils, and so on [28]. People who are prone to take dietary supplements usually take more care about their health (healthy diet, exercise more, visiting doctors on a regular basis) and believe that supplement use is a part of an overall approach to a healthy lifestyle [29]. Although supplements are available on the market, it is always recommended to acquire vitamins and minerals from natural sources, such as fruits and vegetables [30]. Due to its high content of minerals and other nutrients, fresh WGJ is commonly used as a natural dietary supplement [31]. We hypothesized that the addition of WGJ to the other examined juices would increase the mineral concentration of those juices, and along with that, improve their nutritional value.

The addition of WGJ increased K concentration in all juices except in beet juice, where K concentration decreased by 9% in comparison to pure beet juice (Table 4).

The highest increase in K concentration, after the addition of WGJ, was measured in apple (2.18-fold), lemon (60%), and orange (37%) juice in comparison to pure juices. In general, the highest increase after the addition of WGJ in comparison to pure juices was obtained for Ca concentration. For example, Ca concentration in apple juice, after the addition of WGJ increased 12-fold, while in lemon, carrot, and orange juice, the increase in Ca concentration was 3.26, 3.18, and 2.52-fold, respectively, compared to pure juices. A significant increase in Mg concentration was obtained in apple (6.81-fold), carrot (3.92-fold), lemon (2.37-fold), and orange (89%) juices after WGJ addition.

As far as microelements are concerned, after the addition of WGJ to fruit and vegetable juices, the largest increase was obtained for Mn concentration. A Mn concentration increased 24-fold in apple and 22-fold in orange juice, while in beet and carrot juice, the increase was 4- and 3.9-fold, respectively, in comparison to Mn concentration in pure juices. After the addition of WGJ, Fe concentration in apple juice increased 12.2-fold. In carrot juice, the increment was 1.9-fold, while in beet juice, a slight increase in Fe was found (14%).

Zn concentration in apple (4.2-fold), orange (3.8-fold), and carrot juice (1.9-fold) increased significantly, while in beet juice, only a small increase was obtained (0.4%) after the addition of WGJ (Table 5). WGJ showed potential as a fortifier of mineral concentrations of other juices when mixed with them. Furthermore, mixing WGJ with other juices could neutralize the smell and taste of WGJ, which many find unpalatable. So far, consumers preferred wheatgrass beverages containing a mixture of banana and guava in comparison to pure wheatgrass juice or wheatgrass beverages containing lemon and strawberries [32].

#### 3.1.3. The Effect of Lemon Juice Addition on In Vitro Bioaccessibility of Macro and Microelements in Different Fruit and Vegetable Juices

Vitamin C has substantial antioxidant properties that play an important role in disease prevention [33,34,35]. Furthermore, vitamin C affects the absorption of some minerals. The role of vitamin C in Fe absorption is well researched, and it is well known that vitamin C, as well as other organic acids, enhances Fe(II) and Fe(III) uptake in a human organism [36]. On the other hand, the role of vitamin C in the absorption of other macro- and microelements is not completely elucidated yet, and there are confronting results about the role of vitamin C in mineral absorption [37,38,39,40]. In this research, we hypothesized that the addition of lemon juice, as a natural source of vitamin C, will affect the bioaccessibility of some minerals in fruit and vegetable juices.

**Figure 1:**
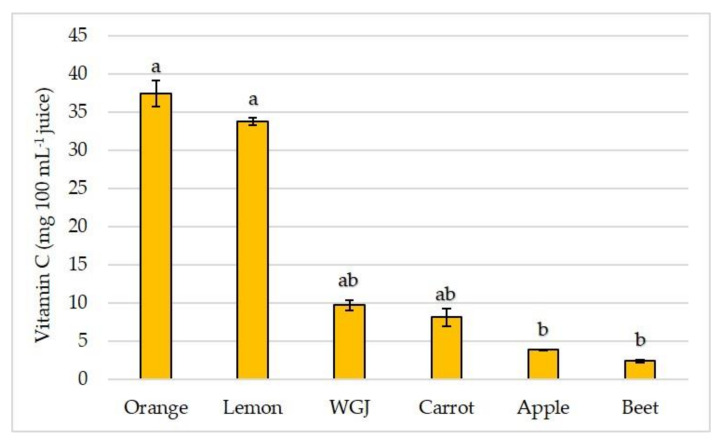
Vitamin C concentration (mg 100 mL^−1^ juice) in wheatgrass (WGJ), apple, beet, carrot, orange, and lemon juice. Values are reported as mean ± SEM, *n* = 3. Means followed by different letters are statistically significantly different at *p* < 0.05 by Kruskal-Wallis test.

Fresh fruit, vegetable, and WGJ were tested with an aim to obtain vitamin C concentration. Fruit juices are an important source of vitamin C [41] in an everyday diet, and as it is expected, orange and lemon juice had the highest vitamin C concentrations in comparison to other juices (Figure 1). Lemon juice had the second-highest vitamin C concentration that was almost 14-fold higher than in beet juice, 8.7-fold higher than in apple juice, 4.2-fold higher than in carrot, and 3.5-fold higher vitamin C concentration than in WGJ.

The addition of fresh lemon juice, as a natural source of vitamin C, decreased in vitro bioaccessibility of K, Ca, and Mg in beet and carrot juices (Table 4). The decrease ranged between 0.2% (for Ca in carrot juice) and 27% (for K in beet juice). On the other hand, in apple and orange juice percentage of bioaccessibility of K, Ca, and Mg increased after the addition of lemon juice. The smallest increase was obtained for K (3% for orange juice and 5% for beet juice), followed by Mg (5% in orange and 11% in apple juice) and Ca with an increase of 13% and 14% in apple and orange juice, respectively.

In general, lemon juice addition to other juices resulted in a larger increase in the percentage of bioaccessibility of microelements (Mn, Fe, and Zn) in comparison to macro-elements (K, Ca, and Mg). The most substantial increase in microelement percentage of bioaccessibility after the addition of lemon juice was obtained for Fe (95%) and Zn (88%) in apple juice. As a result of lemon juice addition, a rather large increase in Zn bioaccessibility was noted in carrot (23%) and orange juice (20%).

### 3.2. Total Phenolic and Flavonoid Content and Antioxidant Capacity in Fresh Fruit, Vegetable, and Wheatgrass Juice

**Figure 2:**
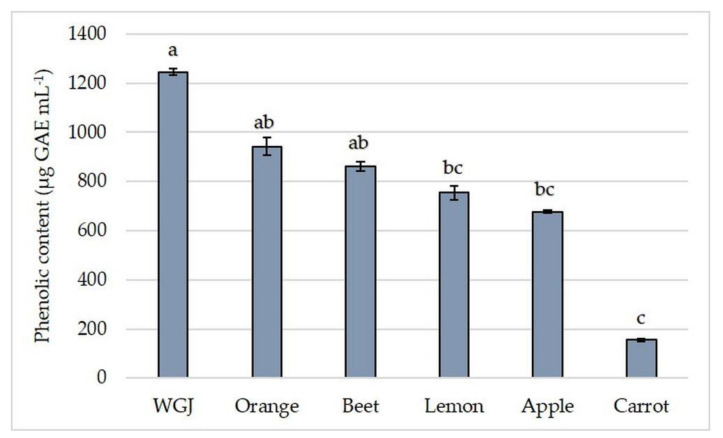
Total phenolic content (µg GAE mL^−1^) in fresh wheatgrass (WGJ), apple, beet, carrot, orange, and lemon juice. Values are reported as mean ± SEM, *n* = 3. Means followed by different letters are statistically significantly different at *p* < 0.05 by Kruskal-Wallis test.

Fruit and vegetable juices are considered a good source of dietary polyphenols [42]. Besides its antioxidant properties, plant polyphenols showed a positive effect on gut microbiota and a decrease in intestinal inflammation [43]. Examined juices significantly differed in their total phenolic content (Figure 2). A fresh WGJ had the highest total phenolic content in comparison to other juices. Orange and beet juice did not significantly differ from WGJ in phenolic content, yet orange juice had 24% lower, and beet juice had 31% lower total phenolic content than WGJ. The lowest total phenolic content was found in carrot juice, and it was 87% lower than in WGJ (Figure 2).

**Figure 3:**
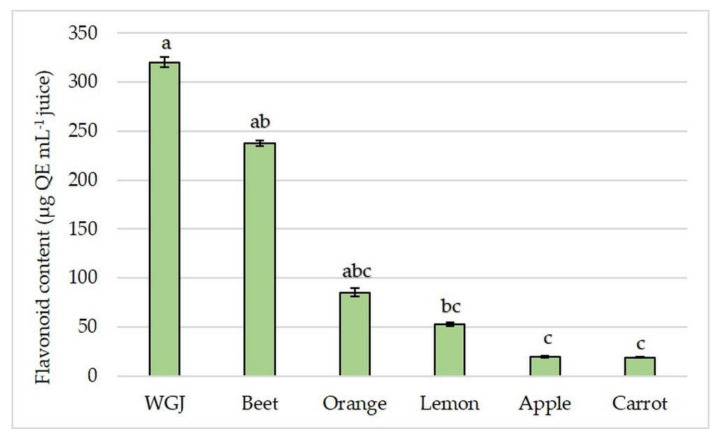
Total flavonoid content (µg QE mL^−1^ juice) in fresh wheatgrass (WGJ), apple, beet, carrot, orange, and lemon juice. Values are reported as mean ± SEM, *n* = 3. Means followed by different letters are statistically significantly different at *p* < 0.05 by Kruskal-Wallis test.

The differences between juices were larger in total flavonoid content than in phenolic content. Again, WGJ had the highest flavonoid content, followed by beet juice that had 26% lower total flavonoid content (Figure 3) compared to WGJ. The lowest flavonoid content was found in apple and carrot juices, and it was 16- and 17-fold lower than in WGJ, respectively.

Polyphenols, including phenolic and flavonoids, are secondary plant metabolites that can protect against the development of chronic diseases, such as diabetes, osteoporosis, cardiovascular diseases, cancers, and neurodegenerative diseases [44,45]. The ability of plant polyphenols to scavenge free radicals and inactivate other pro-oxidants classifies them among the most powerful antioxidants [46,47,48]. Antioxidant activity of fruit, vegetable, and WGJ was assessed by measuring DPPH radical scavenging activity.

**Figure 4:**
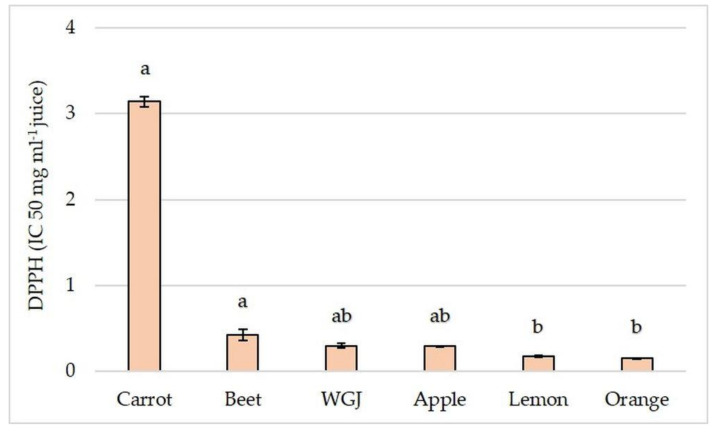
Antioxidant activity measured by DPPH radical scavenging activity. Values are reported as mean ± SEM, *n* = 3. Means followed by different letters are statistically significantly different at *p* < 0.05 by the Kruskal–Wallis test.

As was expected, juices that had higher total phenolic and flavonoid content showed higher antioxidant activity (Figure 4) since total phenolic and flavonoid content is correlated to radical scavenging antioxidant activity [49]. Although WGJ had the highest total phenolic content in comparison to other juices, the addition of WGJ to other juice resulted in a significant increase in the total phenolic only in carrot juice (Table 6) where total phenolic increased 4.1-fold after the addition of WGJ. The total flavonoid content in apple, carrot, orange, and lemon juice increased significantly after the addition of WGJ (Table 6). The largest increase was obtained in carrot juice (8.9-fold), followed by apple (7.2-fold), lemon (2.2-fold), and orange (1.5-fold) juice.

## 4. Conclusions

A total and in vitro bioaccessible concentrations of K, Ca, Mg, Mn, Fe, and Zn, vitamin C concentration, total phenolic and flavonoid content, and antioxidant activity in apple, beet, carrot, orange lemon, and WGJ were studied. A WGJ had the highest total and bioaccessible concentrations of Ca, Mg, Mn, Fe, and Zn in comparison to other examined juices, and WGJ proved to be a rich source of minerals. In general, the addition of WGJ to other juices increased total and bioaccessible concentrations of elements in all examined juices. The exception was K in beet and Fe concentrations in orange juice, which decreased after the addition of WGJ. Furthermore, the addition of lemon juice, as a source of vitamin C, increased bioaccessibility of Mn, Fe, and Zn in all juices (exception was Zn bioaccessibility in beet juice that decreased by 3%), and increased bioaccessibility of K, Ca, and Mg in apple and orange juice. On the other hand, the addition of lemon juice decreased the bioaccessibility of K, Ca, and Mg in beet and carrot juice. The highest total phenolic and flavonoid content was found in WGJ. The addition of WGJ to other juices resulted in an increase in phenolic content in all juices, but only in carrot juice was the increase statistically significant. Flavonoid content in apple, carrot, orange, and lemon juice significantly increased after the addition of WGJ. Obtained results indicated that WGJ is a good source of minerals and antioxidants. A WGJ in a mixture with fruit and vegetable juice can enhance their nutritional value, while the addition of lemon juice can increase the bioaccessibility of some minerals. Since the bioaccessibility of minerals is a complex trait, further research of mineral interaction during absorption and the role of different antinutrients should be further investigated.

## Figures and Tables

**Table 1:** Juices and mixtures of juices used in the experiment.

Juice	Volume Used in Experiment
Wheatgrass (WGJ)	10 mL juice
Apple	
Orange	
Beet	
Carrot	
Lemon	
Apple + WGJ	6 mL juice + 4 mL WGJ
Orange + WGJ	
Beet + WGJ	
Carrot + WGJ	
Lemon + WGJ	
Apple + WGJ + Lemon	5.75 mL juice + 3.75 mL WGJ + 0.5 mL lemon
Orange + WGJ + Lemon	
Beet + WGJ + Lemon	
Carrot + WGJ + Lemon	

**Table 2:** Total and bioaccessible concentrations of K, Ca, and Mg in wheatgrass (WGJ), apple, beet, carrot, orange, and lemon juice.

Total Concentrations (mg L^−1^)				Bioaccessible Concentrations (mg L^−1^)		
	K	Ca	Mg	K	Ca	Mg
WGJ	3262 ± 66.6 b	431 ± 1.28 a	325 ± 3.65 a	2828 ± 19.1 b	305 ± 1.94 a	248 ± 0.43 a
Apple	806 ± 75.5 e	14.4 ± 1.71 d	21.0 ± 0.81 c	585 ± 18.0 d	0.00 *	11.0 ± 0.52 c
Beet	4067 ± 187.3 a	30.3 ± 0.55 d	286 ± 19.7 a	3908 ± 217.6 a	3.66 ± 0.08 d	218 ± 14.05 a
Carrot	2109 ± 126.5 c	59.4 ± 0.96 c	33.4 ± 1.48 c	1845 ± 88.0 c	16.9 ± 0.85 c	20.5 ± 11.5 c
Orange	1620 ± 156 cd	90 ± 7.65 b	100 ± 12.2 b	1177 ± 84.2 d	40.1 ± 1.70 b	69.2 ± 7.66 b
Lemon	1257 ± 73.1 de	63.5 ± 1.32 c	69.2 ± 4.02 bc	1097 ± 36.8 d	34.9 ± 3.32 b	49.9 ± 4.70 bc
F value	102.92	2238.96	185.29	145.6	4511.88	227.79
*p*	<0.0001	<0.0001	<0.0001	<0.0001	<0.0001	<0.0001

Values are reported as mean ± SEM, *n* = 3. F and *p*-values were obtained from one-way ANOVA. Means followed by different letters are statistically significantly different at *p* < 0.01 by Tukey HSD test. * under the detection limit.

**Table 3:** Total and bioaccessible concentrations of Mn, Fe, and Zn in wheatgrass (WGJ), apple, beet, carrot, orange, and lemon juice.

Total Concentrations (mg L^−1^)				Bioaccessible Concentrations (mg L^−1^)		
	Mn	Fe	Zn	Mn	Fe	Zn
WGJ	6.40 ± 0.07 a	4.64 ± 0.11 a	2.80 ± 0.03 a	4.93 ± 0.09 a	2.66 ± 0.08 a	1.05 ± 0.02 b
Apple	0.108 ± 0.01 c	0.149 ± 0.02 d	0.303 ± 0.07 c	0.075 ± 0.007 c	0.00 *	0.13 ± 0.016 e
Beet	0.72 ± 0.001 b	3.04 ± 0.01 b	2.75 ± 0.11 a	0.13 ± 0.004 bc	1.27 ± 0.13 b	1.43 ± 0.04 a
Carrot	0.70 ± 0.02 b	1.64 ± 0.01 c	0.80 ± 0.07 b	0.35 ± 0.03 b	1.20 ± 0.03 b	0.58 ± 0.04 c
Orange	0.115 ± 0.002 c	2.57 ± 0.17 b	0.34 ± 0.02 c	0.093 ± 0.01 c	0.43 ± 0.04 c	0.32 ± 0.02 d
Lemon	0.099 ± 0.01 c	0.36 ± 0.03 d	0.57 ± 0.06 bc	0.083 ± 0.01 c	0.18 ± 0.00 cd	0.38 ± 0.04 d
F value	6260.78	400.48	420.82	2740.94	223.4	266.92
*p*	<0.0001	<0.0001	<0.0001	<0.0001	<0.0001	<0.0001

Values are reported as mean ± SEM, *n* = 3. F and *p*-values were obtained from one-way ANOVA. Means followed by different letters are statistically significantly different at *p* < 0.01 by Tukey HSD test. * under the detection limit.

**Table 4:** Effect of wheatgrass juice (WGJ) and lemon juice addition on total and bioaccessible concentrations of K, Ca, and Mg in apple, beet, carrot, orange, and lemon juice.

	Total Concentrations (mg L^−1^)			Bioaccessible Concentrations (mg L^−1^)		
	K	Ca	Mg	K	Ca	Mg
Apple	806 ± 75.5 b	14.4 ± 1.71 b	21.0 ± 0.81 b	585 ± 18.0 b	0.00 b *	11.0 ± 0.52 b
Apple + WGJ	1760 ± 56.4 a	181 ± 3.97 a	143 ± 2.65 a	1486 ± 70.7 a	116 ± 12.3 a	104 ± 2.94 a
Apple + WGJ + Lemon	1679 ± 28.5 a	165 ± 3.10 a	129 ± 4.11 a	1489 ± 54.6 a	120 ± 8.2 a	104 ± 5.45 a
F value	88.02	900.69	542.35	98.13	63.62	224,48
*p*	<0.0001	<0.0001	<0.0001	<0.0001	<0.0001	<0.0001
Beet	4067 ± 187.3	165 ± 3.10 b	286 ± 19.7	3908 ± 217.6 a	3.66 ± 0.08 b	218 ± 14.05
Beet + WGJ	3683 ± 79.0	182 ± 2.10 a	292 ± 25.4	3240 ± 191.6 ab	40.7 ± 2.13 a	221 ± 6.44
Beet + WGJ + Lemon	3584 ± 83.7	175 ± 2.65 a	275 ± 21.7	2296 ± 106.9 b	33.8 ± 1.77 a	202 ± 12.7
F value	4.03	1873.39	0.16	20.61	151.71	0.16
*p*	0.0776	<0.0001	0.8577	0.0021	<0.0001	0.8577
Carrot	2109 ± 126.5	59.4 ± 0.96 b	33.4 ± 1.48 b	1845 ± 88.0	16.9 ± 0.85 b	20.5 ± 11.5 b
Carrot + WGJ	2496 ± 70.3	189 ± 14.1 a	131 ± 11.7 a	2319 ± 22.6	124 ± 13.6 a	112 ± 11.8 a
Carrot + WGJ + Lemon	2504 ± 25.6	194 ± 4.99 a	140 ± 6.05 a	2082 ± 162.6	127 ± 114.8 a	105 ± 18.1 a
F value	7.09	78.15	229.41	4.85	50.64	59.14
*p*	0.0263	<0.0001	0.0001	0.0559	0.0002	0.0001
Orange	1620 ± 155.7	90 ± 7.65 b	100 ± 12.2 b	1177 ± 84.2	40.1 ± 1.70 b	69.2 ± 7.66 b
Orange + WGJ	2227 ± 82.3	227 ± 1.15 a	189 ± 7.33 a	1751 ± 84.8	161 ± 0.16 a	137 ± 3.00 a
Orange + WGJ + Lemon	2135 ± 111.0	202 ± 4.05 a	170 ± 10.36 a	1721 ± 125.7	164 ± 2.00 a	130 ± 2.04 a
F value	7.42	1783.21	21.26	10.42	210.02	229.41
*p*	0.0239	<0.0001	0.0019	0.0112	<0.0001	0.0001
Lemon	1257 ± 73.1 b	63.5 ± 1.32 b	69.2 ± 4.02 b	1097 ± 36.8 b	34.9 ± 3.32 b	49.9 ± 4.70 b
Lemon + WGJ	2019 ± 17.4 a	207 ± 3.12 a	164 ± 4.77 a	1786 ± 74.5 a	151 ± 2.27 a	123 ± 0.50 a
F value	102.57	1783.21	21.26	68.8	834.85	236.99
*p*	0.0005	<0.0001	0.0019	0.0012	<0.0001	0.0001

Values are reported as mean ± SEM, *n* = 3. F and *p*-values were obtained from one-way ANOVA. Means followed by different letters are statistically significantly different at *p* < 0.01 by Tukey HSD test. * under the detection limit.

**Table 5:** Effect of wheatgrass juice (WGJ) and lemon juice addition on total and bioaccessible concentrations of Mn, Fe, and Zn in apple, beet, carrot, orange, and lemon juice.

	Total Concentrations (mg L^−1^)			Bioaccessible Concentrations (mg L^−1^)		
	Mn	Fe	Zn	Mn	Fe	Zn
Apple	0.11 ± 0.01 b	0.15 ± 0.02 b	0.30 ± 0.07 b	0.08 ± 0.007 b	0.00 *	0.13 ± 0.02 c
Apple + WGJ	2.63 ± 0.067 a	1.83 ± 0.01 a	1.26 ± 0.00 a	2.29 ± 0.02 a	0.41 ± 0.02 b	0.52 ± 0.02 b
Apple + WGJ + Lemon	2.46 ± 0.069 a	1.69 ± 0.06 a	1.16 ± 0.01 a	2.32 ± 0.04 a	0.74 ± 0.08 a	0.90 ± 0.01 a
F value	638.08	688.43	855.57	2367.13	56.8	855.57
*p*	<0.0001	<0.0001	<0.0001	<0.0001	0.0001	<0.0001
Beet	0.72 ± 0.001 b	3.04 ± 0.01	2.75 ± 0.11	0.13 ± 0.004 b	1.27 ± 0.13	1.43 ± 0.04
Beet + WGJ	2.89 ± 0.04 a	3.46 ± 0.18	2.76 ± 0.03	1.93 ± 0.10 a	1.69 ± 0.08	1.43 ± 0.07
Beet + WGJ + Lemon	2.73 ± 0.05 a	3.20 ± 0.11	2.57 ± 0.02	2.02 ± 0.13 a	1.62 ± 0.11	1.29 ± 0.18
F value	937.99	3.11	2.53	129.66	4.38	0.52
*p*	<0.0001	0.1185	0.1595	<0.0001	0.0671	0.6167
Carrot	0.70 ± 0.02 b	1.64 ± 0.01	0.80 ± 0.07 b	0.35 ± 0.03 b	1.20 ± 0.03 b	0.58 ± 0.04
Carrot + WGJ	2.76 ± 0.29 a	3.17 ± 0.44	1.51 ± 0.08 a	2.06 ± 0.03 a	1.81 ± 0.06 a	0.78 ± 0.06
Carrot + WGJ + Lemon	2.85 ± 0.04 a	2.73 ± 0.09	1.51 ± 0.01 a	2.45 ± 0.12 a	1.85 ± 0.13 a	0.96 ± 0.08
F value	51.92	9.38	43.68	250.8	18.46	9.83
*p*	0.0002	0.0142	0.0003	0.0002	0.0027	0.0128
Orange	0.12 ± 0.002 b	2.57 ± 0.17	0.34 ± 0.02 b	0.09 ± 0.01 b	0.43 ± 0.04 b	0.32 ± 0.02 b
Orange + WGJ	2.63 ± 0.02 a	1.95 ± 0.01	1.28 ± 0.03 a	2.32 ± 0.06 a	1.49 ± 0.07 a	0.94 ± 0.04 a
Orange + WGJ + Lemon	2.39 ± 0.08 a	1.83 ± 0.13	1.18 ± 0.06 a	2.15 ± 0.10 a	1.44 ± 0.11 a	1.04 ± 0.02 a
F value	964.85	10.37	160.61	315.64	56.68	174.48
*p*	<0.0001	0.0113	<0.0001	<0.0001	0.0001	<0.0001
Lemon	0.10 ± 0.01 b	0.36 ± 0.03 b	0.57 ± 0.06 b	0.08 ± 0.01 b	0.18 ± 0.00 b	0.38 ± 0.04 b
Lemon + WGJ	2.66 ± 0.082 a	2.01 ± 0.04 a	1.38 ± 0.01 a	2.28 ± 0.03 a	1.47 ± 0.04 a	1.07 ± 0.02 a
F value	757.72	1049	209.28	3878.23	1103.83	222.8
*p*	<0.0001	<0.0001	0.0001	<0.0001	<0.0001	0.0001

Values are reported as mean ± SEM, *n* = 3. F and *p*-values were obtained from one-way ANOVA. Means followed by different letters are statistically significantly different at *p* < 0.01 by Tukey HSD test. * under the detection limit.

**Table 6:** Effect of wheatgrass (WGJ) and lemon juice addition on the concentration of vitamin C (mg 100 mL^−1^ juice), total phenolic content (µg GAE mL^−1^), total flavonoid content (mg l^−1^), and DPPH (IC 50 mg mL^−1^ juice) in apple, beet, carrot, orange, and lemon juice.

	Vitamin C	Phenolic Content	Flavonoid Content	DPPH
Apple	3.93 ± 0.024 b	677.38 ± 4.32	19.88 ± 0.91 b	0.29 ± 0.002
Apple + WGJ	25.47 ± 0.787 ab	826.82 ± 11.03	143.77 ± 7.74 a	0.34 ± 0.018
Apple + WGJ + Lemon	36.40 ± 0.279 a	858.65 ± 41.34	90.11 ± 2.28 ab	0.31 ± 0.005
Beet	2.46 ± 0.174 b	861.27 ± 17.52	237.68 ± 2.73	0.42 ± 0.066
Beet + WGJ	16.73 ± 0.627 ab	1005.47 ± 10.81	244.78 ± 6.83	0.30 ± 0.023
Beet + WGJ + Lemon	30.96 ± 0.858 a	961.02 ± 34.32	141.41 ± 4.56	0.22 ± 0.003
Carrot	8.15 ± 1.135 b	154.53 ± 5.67 b	18.83 ± 0.53 b	3.14 ± 0.061 a
Carrot + WGJ	27.12 ± 0.881 ab	634.68 ± 23.23 ab	169.02 ± 0.46 a	0.49 ± 0.034 ab
Carrot + WGJ + Lemon	37.13 ± 0.733 a	796.48 ± 12.11 a	92.48 ± 1.82 ab	0.35 ± 0.005 b
Orange	37.41 ± 1.726	942.17 ± 36.98	85.38 ± 4.1 b	0.15 ± 0.006
Orange + WGJ	44.66 ± 2.436	1122.32 ± 31.07	128.78 ± 6.38 a	0.22 ± 0.020
Orange + WGJ + Lemon	44.90 ± 1.906	1033.93 ± 63.84	100.37 ± 0.91 ab	0.21 ± 0.006
Lemon	33.79 ± 0.461	754.28 ± 28.24	53.03 ± 1.82 b	0.18 ± 0.008
Lemon + WGJ	45.15 ± 1.095	1020.82 ± 7.14	115.37 ± 4.1 a	0.25 ± 0.010

Values are reported as mean ± SEM, *n* = 3. Means followed by different letters are statistically significantly different at *p* < 0.05 by Kruskal–Wallis test (adjusted by the Bonferroni correction for multiple tests).

## Data Availability

The datasets generated for this study are available on request to the corresponding author.
